# Socio-ecological connectivity differs in magnitude and direction across urban landscapes

**DOI:** 10.1038/s41598-020-61230-9

**Published:** 2020-03-06

**Authors:** Monika Egerer, Nakisha Fouch, Elsa C. Anderson, Mysha Clarke

**Affiliations:** 1 Department of Environmental Studies, University of California, Santa Cruz, CA USA; 2 Department of Ecology, Ecosystem Sciences/Plant Ecology, Technische Universität Berlin, Rothenburgstrasse 12, Berlin, 12165 Germany; 3 Department Wildlife and Fisheries Biology, Clemson University, Clemson, SC USA; 4 Department of Biological Sciences, University of Illinois at Chicago, Chicago, IL USA; 5 Cary Institute of Ecosystem Studies, Millbrook, NY USA; 6 Department of Geography and the Environment, Villanova University, Villanova, PA USA; 7 School of Forest Resources and Conservation, University of Florida, Gainesville, FL USA

**Keywords:** Urban ecology, Environmental sciences

## Abstract

Connectivity of social-ecological systems promotes resilience across urban landscapes. Community gardens are social-ecological systems that support food production, social interactions, and biodiversity conservation. We investigate how these hubs of ecosystem services facilitate socio-ecological connectivity and service flows as a network across complex urban landscapes. In three US cities (Baltimore, Chicago, New York City), we use community garden networks as a model system to demonstrate how biophysical and social features of urban landscapes control the pattern and magnitude of ecosystem service flows through these systems. We show that community gardens within a city are connected through biological and social mechanisms, and connectivity levels and spatial arrangement differ across cities. We found that biophysical connectivity was higher than social connectivity in one case study, while they were nearly equal in the other two. This higher social connectivity can be attributed to clustered distributions of gardens within neighborhoods (network modularity), which promotes neighborhood-scale connectivity hotspots, but produces landscape-scale connectivity coldspots. The particular patterns illustrate how urban form and social amenities largely shape ecosystem service flows among garden networks. Such socio-ecological analyses can be applied to enhance and stabilize landscape connectedness to improve life and resilience in cities.

## Introduction

Urban landscapes are social-ecological systems that are growing in geographic area and population density across much of the world^1,2^. Indeed, nearly two-thirds of the world’s population will live in cities by 2050^3^, with 89% projected for the USA alone. This forecast is prompting discussion on the sustainability of urban growth, and the maintenance of social well-being^4^ and environmental integrity in urban landscapes^5^.

The interactions between social and biophysical features in cities regulate ecosystem functions^1,5–8^ which provides essential ecosystem services to urban populations^9^. Ecosystem services include supporting services (e.g. nutrient cycling), provisioning services (e.g. food), regulating services (e.g. climate regulation), and cultural services (e.g. recreation)^10^. These services enhance the well-being of urban residents by improving physical and mental health^11^, and providing the basic materials for a good life that allow people freedom and choice of action^10^. Yet the maintenance and management of ecosystem services across urban landscapes is challenging because of high spatial heterogeneity in land use composition and structure^12^, and the high social diversity in demographics and resource access among populations^13–15^. Urban ecosystem services that support human well-being are thus derived from multiple and diverse biophysical, social, technological, and economic features that vary with spatial scale^16^ across the landscape^17,18^.

Biophysical and social heterogeneity of landscapes determines the connectivity of ecosystem services through the facilitation or resistance of ecosystem service flows^19–21^. Landscape facilitation of flows occurs when, for example, organisms that provide ecosystem services (e.g., pollinators, pest control agents) can easily move among habitats across a landscape, meaning that habitats are more connected through these service providers. Landscape resistance occurs when barriers exist to movement. Ecosystem services are thus better facilitated when there is enhanced landscape connectivity that facilitates species movement for migration, pollination, dispersal or interactions underlying ecosystem services. Connectivity as a socio-ecological principle is “the way and degree to which resources, species, or social actors disperse, migrate or interact across ecological and social landscapes”^22,23^. Biophysical connectivity generally relates to how the biophysical features of the landscape facilitate the movement of organisms between resource patches in the context of a management objective^24^. Social connectivity relates to how increased information sharing and connection among people within communities enhances the spread of ideas, and improves governance structures and human well-being in society^25–27^. For example, Latino community gardens in New York City provide a space for participants to share information about cultural heritage, voter registration, identity and food^28^. While social and biophysical connectivity can individually provide insight into how a particular system functions, synthesizing these two domains to integrate human behaviors and activities with ecological processes is of particular interest in human-dominated ecosystems^29^.

Connectivity of urban landscapes has concrete social-environmental outcomes. Higher biophysical connectivity of green infrastructure^30^ can increase the mobility of animals between habitat patches across the physical landscape (e.g., vegetatively diverse green roofs designed to facilitate the connectivity of beneficial bees and natural predators^31^). Higher social connectivity can improve distribution of information and resources across the social landscape^22,32^ to strengthen social networks, public health, and community robustness^4,33^. On the other hand, low connectivity due to high resistance – the hindrance of movement across a landscape – caused by biophysical features (e.g. lack of green spaces) or social features (e.g. lack of community centers) of the landscape may be associated with environmental degradation, biodiversity loss, and social isolation that impact ecosystem function and human well-being^34–36^. Low connectivity can compromise ecosystem service delivery across landscapes and decreases the resilience^20^ – the capacity of the system to tolerate and respond to disturbance and social-environmental change^37^ – of social-ecological systems^22^. Therefore, it is critical to support and bolster ecosystem services in the face of disturbance and continuous change to promote urban resilience^38^.

Improving social and ecological connectivity through landscape design is of growing interest to city planners, urban conservationists, and scientists because of potential positive effects on urban resilience under environmental change including climate change, urbanization, biodiversity loss^5,39–42^. For example, adding more urban greenways can improve ecological connectivity by promoting biodiversity and its movement, thereby potentially bolstering resilience^43^. Incorporating new social spaces fosters cohesion and well-being of urban dwellers who use those spaces^44–47^. Yet the relationship between connectivity and ecosystem resilience is likely non-linear and complex because of urban social and environmental heterogeneity^48^, which may drive ecosystem function loss or service trade-offs^49^. For example, modularity, or the clustering of internally well-connected forms of green infrastructure may be more important to ecosystem service provision and urban resilience than green infrastructure distributed across an urban landscape (e.g., urban trees species connectivity in relation to pests; networks of bioswales)^50^. Thus, the connection between connectivity and urban resilience is certainly city and system-network dependent, and requires further investigation in different contexts to translate into urban planning. To inform sustainable and resilient cities, we need to better understand how networks of social-ecological systems can facilitate the connectivity of ecosystem service flows among systems within the network in relation to biophysical and social landscape features^18^.

Urban community gardens are multifunctional social-ecological systems distributed across city neighborhoods that are sources of ecosystem service flows^51^. As ecological systems, gardens serve as habitats for biodiversity^52–54^, regulate climate^55,56^, and control stormwater runoff^51^. As social systems, community gardens increase fresh food access^57,58^ and security^59^, improve social networks^47,60^, provide education and social learning^61–64^, and increase community organizing and self-empowerment^45,47,62,65^. In sum, gardens are simultaneously sites of environmental processes, social connectivity, and human well-being^66^. The social and ecological functions that community gardens serve make them “nodes” of ecosystem service bundles in the landscape of ecosystem services flows^67^ that in turn benefit gardeners and surrounding local communities^66,68^. There is little understanding of the role of garden networks in ecosystem service connectivity across cities, yet this knowledge could benefit efforts to build urban resilience to environmental and social disturbances.

From a landscape perspective, interactions among community gardens within a network can enhance ecosystem service flows by connecting biological corridors and facilitating human social interactions^53,69^. Despite the varied benefits of community gardens, they are often not evenly dispersed across urban landscapes and can exhibit clustering within certain neighborhoods^70^ as with other green spaces^14^. Similarly, the connectivity of ecosystem services vary across heterogeneous landscapes, contributing to socio-environmental justice concerns^71^. Measuring the connectivity of flows across community garden networks in relation to community garden locations in the landscape could inform stakeholder’s strategic establishment of gardens in areas to increase landscape-scale socio-ecological connectivity and service provision within their given network^72^. Yet connectivity assessments often do not consider both biophysical and social features of urban landscapes to inform decision making^73^. It is thus essential to develop new approaches to assess both social and biophysical connectivity to create future applicable strategies that bolster the connectivity of social-ecological system networks and thus their resilience in changing environments^61^.

In this study, we quantified the magnitude of biophysical, social, and combined socio-ecological connectivity across community garden networks using resistance landscapes based on the biophysical and social features of three cities. We utilized community gardens as a model social-ecological system because gardens are a major confluence of ecological and social processes, and thus nodes of ecosystem service flows. Here we used a stakeholder-informed approach to map community garden nodes within a network across a city landscape to examine how levels of socio-ecological connectivity and flow of ecosystem services are supported through these stakeholder networks, where stakeholder activity is a de facto steward of ecosystem services. We did this for three US cities (Baltimore, Chicago and New York City) that have a history of urban agriculture^72^ and that are working to add multifunctional green spaces to improve their resilience to current and forecasted social and environmental change^74–76^. We leveraged circuit theory grounded in functional landscape movement ecology to measure levels of socio-ecological resistance to connectivity across garden networks in relation to the biophysical and social features unique to each landscape (Table 1). Circuit theory models the flow dynamics in the context of landscape configuration. Landscape configuration, particularly in urban settings, provides knowledge about the different land covers (biophysical perspective) and demographics (social perspective) and the varying levels of suitability, permeability, purpose, and utilization^31,77,78^. Borrowing from the principles of electrical circuit theory, we are able to leverage habitat patches and features in the landscape to consider more than the existence or lack of existence of a link between patches, but to also consider the resistance to that connection in terms of function and suitability of the principle land cover or demographic types and potential corridors^79^. This flexibility, coupled with the ability to evaluate multiple pathways and variable connectivity probabilities, adds value in using circuit theory for urban habitats where residents (e.g. species or citizens) are assumed more resilient to stress and disturbance but biophysically still require some degree of connectivity from, for example, canopy, parks and green infrastructure^80,81^. To supplement connectivity, we also utilized hotspot analysis to statistically evaluate the spatial pattern of the gardens to better understand their existing and potential influence in the context of the connectivity surface. Hotspot analysis relies less on specificity and predicted measures more often available when evaluating a particular species, but on statistically significant and known spatial relationships^82^. Significant spatial clusters of gardens and their proximity to other clusters were identified in order to verify areas where gardens are commonly located (hotspot) or tend toward isolation (coldspot). Hotspot analysis also incorporates the idea of modularity into the analysis, by showing where significant clustering of gardens and ecosystem flows occurs within the network. The circuit theory methodology allows for predicting continuous network flow connectivity by incorporating both landscape structure and function in measuring all potential movement in relation to resistance, while hotspot analysis informs applicable strategic planning to boost connectivity or resilience in the network by showing where either distributed or modular connectivity patterns occur in relation to study system context. Thus while most connectivity analyses use coarse-scale simplified approaches or focus on single species’ movement^83^, we take a fine-scale socio-ecological approach and include landscape biophysical and social features to show: (**1**) how gardens within a stakeholder network are connected across urban landscapes through biological and social mechanisms; (**2**) how connectivity differs in spatial arrangement, magnitude, and direction between different cities; and (**3**) where there are connectivity hotspots or coldspots in the landscape network to inform strategic urban planning.

**Table 1:** Relevant electrical terms used in circuit theory and the ecological interpretation in relation to landscape movement described by McRae *et al*.^79^, and the social interpretation in this paper from the literature^28^.

Electrical Term	Ecological Interpretation	Social Interpretation
***Resistance***, the opposition that a resistor offers to the flow of electrical current.	Opposition of a habitat type to movement of organisms, similar to ecological concepts of landscape resistance or friction. Grid cells allowing less movement are assigned higher resistance.	Social attributes of e.g. a neighborhood or census tract that prevent community social cohesion and networking or inhibit knowledge exchange are associated with lower well-being indicators.
***Conductance***, inverse of resistance and a measure of a resistor’s ability to carry electrical current.	Analogous to habitat permeability for organisms (e.g. birds, mobile arthropods, mammals). In random-walk applications, it is directly related to the likelihood of a walker choosing to move through a cell or along a graph edge relative to others available to it.	Social attributes that improve distribution of information and resources across people and places to strengthen social networks, public health, and community robustness and social cohesion.
***Current***, flow of charge through a node or resistor in a circuit.	Current flow through nodes (e.g. habitat) or resistors can be used to predict expected net movement probabilities for random walkers (organisms) moving through corresponding graph nodes or edges.	Current flow through nodes (e.g. social spaces) used to predict expected probabilities for random flow (people, social cohesion, knowledge) moving through the landscape.

Our results demonstrate that urban landscape features as well as garden spatial distribution largely shape the nature (direction of biophysical vs. social) and magnitude (intensity) of connectivity across the network, resulting in a unique connectivity profile in each city. Furthermore, we show that areas of high and low connectivity reflect hotspots and coldspots of ecosystem service flows across urban landscapes. Such analyses, we argue, can provide a template for informed urban policy and planning directives for stakeholders aimed at bolstering urban resilience to change^84^.

## Results

### Biophysical, social and socio-ecological connectivity networks

**Figure 1:**
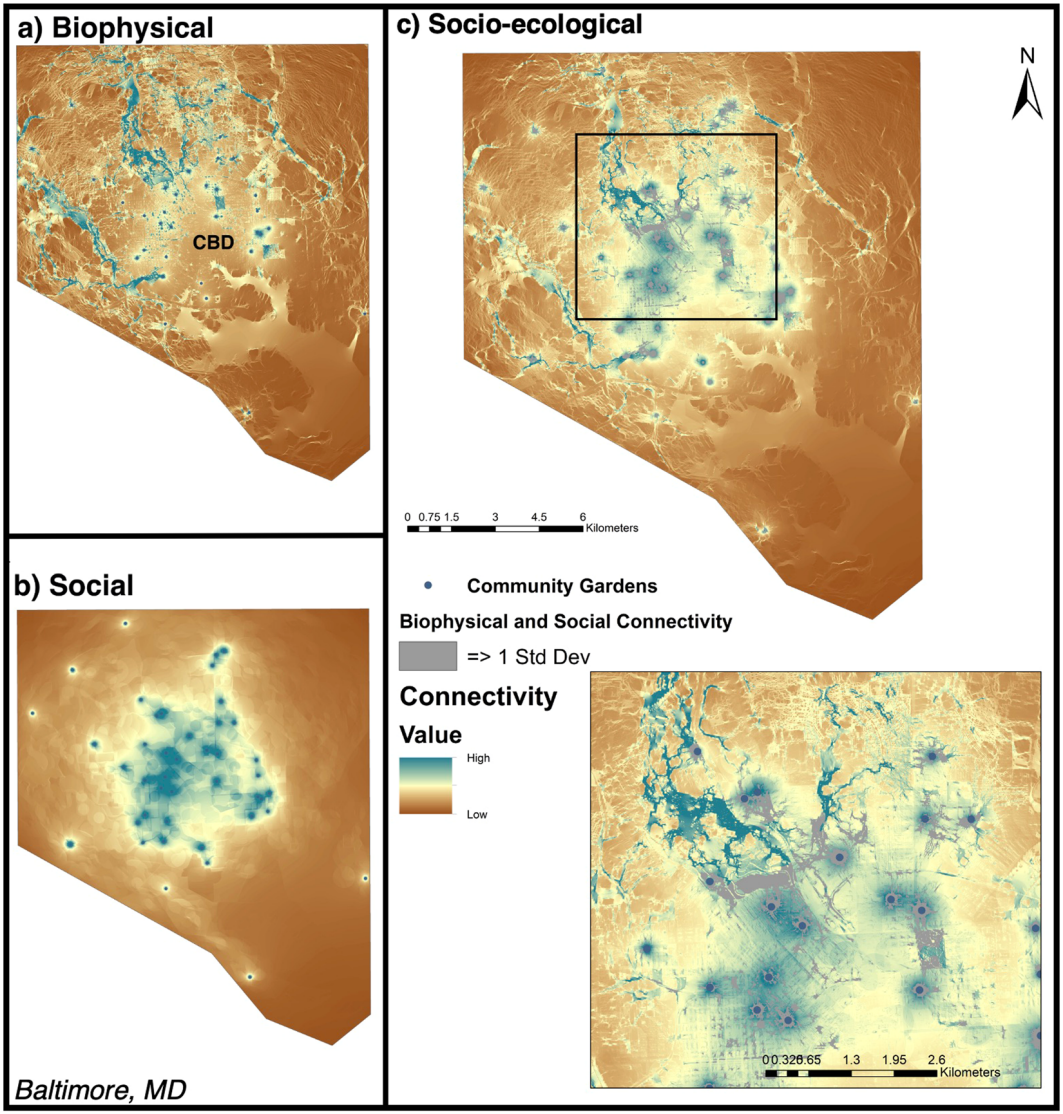
Biophysical (**a**), social (**b**), and additive (socio-ecological; **c**) connectivity models for Baltimore, MD. Inset at finer spatial-scale (black box) provided in panel (c) for more detailed interpretation of connectivity flows. Blue circles represent a node (city-sponsored community garden) used in the connectivity models. Shading gradient from low (brown) to high (blue) represents the highest current flow for biophysical, social, and the two combined; brown areas represent the lowest current flow, respectively. Nodes with no surrounding connectivity had no pairwise match required to generate connectivity models. The approximate geographic location of the Central Business District (CBD) is marked on the map. Areas of both high social and biophysical connectivity are shown in gray shaded areas (**c**). Maps produced using NLCD and NAIP satellite imagery data in ArcGIS (Table 3).

**Figure 2:**
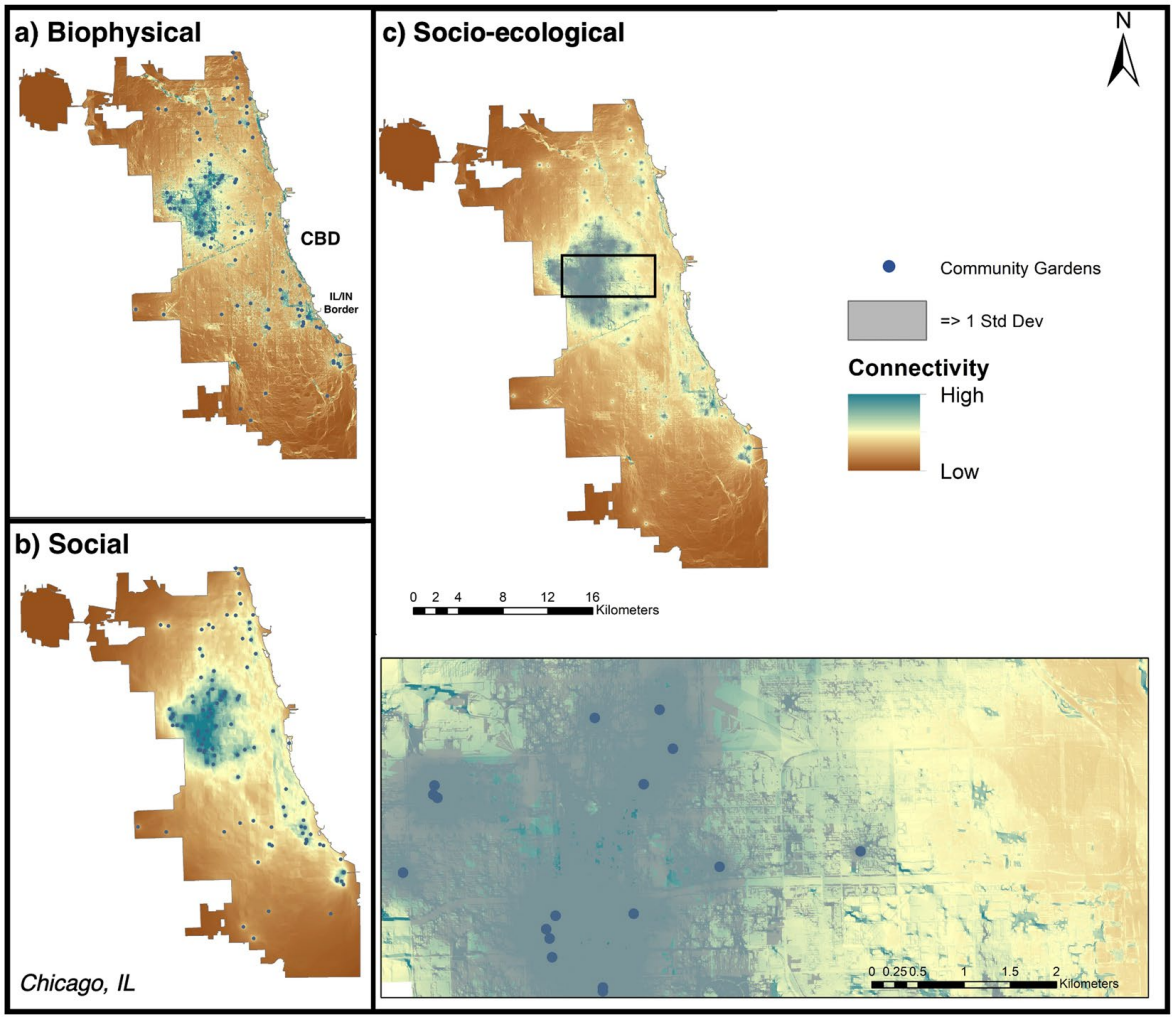
Biophysical (**a**), social (**b**), and additive (socio-ecological; **c**) connectivity models for Chicago, IL. Inset at finer spatial-scale (black box) provided in panel (c) for more detailed interpretation of connectivity flows. Blue circles represent a node (city-sponsored community garden) used in the connectivity models. Shading gradient from low (brown) to high (blue) represents the highest current flow for biophysical, social, and the two combined; brown areas represent the lowest current flow, respectively. Nodes with no surrounding connectivity had no pairwise match required to generate connectivity models. Areas of both high social and biophysical connectivity are shown in gray shaded areas (**c**). To provide relevant context, the approximate geographic location of the Central Business District (CBD) is marked on the map, and the Illinois (IL) and Indiana (IN) border is marked. Maps produced using NLCD and NAIP satellite imagery data in ArcGIS (Table 3).

**Figure 3:**
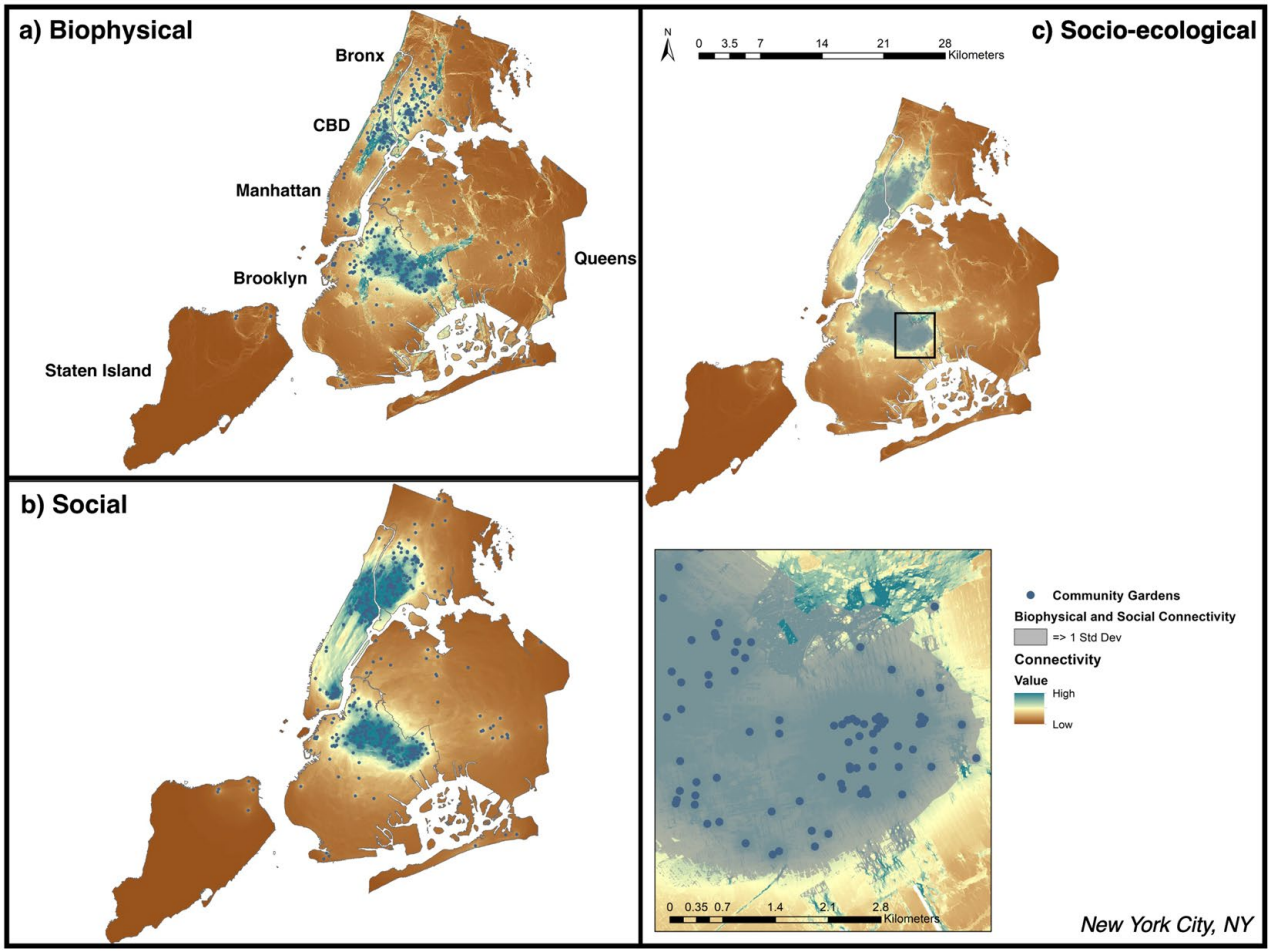
Biophysical (**a**), social (**b**), and additive (socio-ecological; **c**) connectivity models for NYC, NY. Inset at finer spatial-scale (black box) provided in panel (c) for more detailed interpretation of connectivity flows. Blue circles represent a node (city-sponsored community garden) used in the connectivity models. Shading gradient from low (brown) to high (blue) represents the highest current flow for biophysical, social, and the two combined; brown areas represent the lowest current flow, respectively. Nodes with no surrounding connectivity had no pairwise match required to generate connectivity models. Areas of both high social and biophysical connectivity are shown in gray shaded areas (**c**). To provide relevant context, the approximate geographic location of the Central Business District (CBD) is marked on the map, and city boroughs are marked. Maps produced using NLCD and NAIP satellite imagery data in ArcGIS (Table 3).

Community gardens serve as nodes in a network of connectivity relative to the biophysical and social landscape features of each US city, respectively (Figs. 1, 2, 3). The results are weighted current density sums across all pairs of nodes that were included in each city model, appropriate for evaluating landscape-scale connectivity issues. The wide geographic extent of the models provides a landscape perspective for each city of the biophysical and social connectivity derived from specific assumptions (see methods).

Per the construction of our landscape models, all currents originate at the garden nodes and extend through the city in distinct patterns. These patterns can be described as single lines of relatively high current “pinched in” by resistance, or as relatively low sheet currents where no major resistance constrains the flow. Here, the maps show areas of both high and low current flow in the landscape for all connectivity models, highlighting areas where both social and biophysical connectivity were high (Figs. 1–3; Table 2). For all cities, biophysical connectivity initiating from garden nodes radiates pinch-points of high connectivity throughout the landscape. Social connectivity was similarly initiated at garden nodes, but spread across the landscape in a more even sheet-flow.

**Table 2:** Summary statistics (presented as raw median ± standard deviation values) of social, biophysical, and total socio-ecological connectivity for each city.

City	Median social connectivity	Median biophysical connectivity	Median total socio-ecological connectivity	Maximum socio-ecological connectivity
Baltimore	0.118 ± 0.001	0.169 ± 0.001	0.308 ± 0.001	7.890
Chicago	0.172 ± 0.001	0.130 ± 0.001	0.310 ± 0.001	11.620
NYC	0.149 ± 0.0001	0.158 ± 0.001	0.320 ± 0.001	14.560

Because all cities have the same data and theoretical assumptions, we can compare these connectivity values across cities to one another. (Baltimore (N = 43 gardens), Chicago (N = 116), and NYC (N = 476)).

### Differences in connectivity among urban landscapes

**Figure 4:**
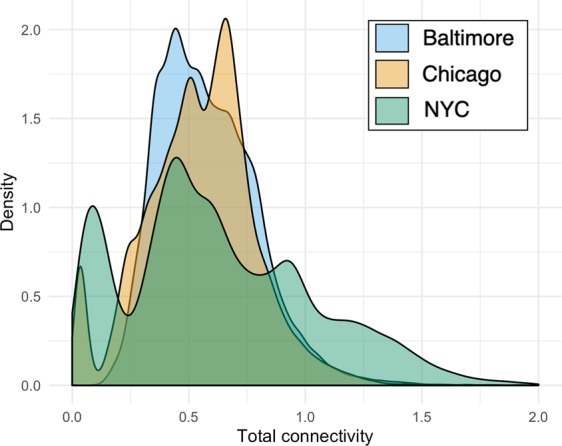
Cumulative density plots for socio-ecological connectivity (summed biophysical and social, square-root transformed for visualization) for Baltimore, Chicago and NYC.

The median normalized connectivity (±SE) for each city revealed that cities differed in total socio-ecological connectivity of these garden networks, and in contributions from biophysical and social connectivity. In Baltimore, total or “socio-ecological” connectivity was highest surrounding nodes located around the city center, while there was moderate connectivity radiating throughout the city. Baltimore had the lowest median and maximum values for socio-ecological connectivity overall, but the distribution was more normal across the city (Figs. 1, 4; Table 2). There are also notable pockets where garden-derived connectivity is absent: along the south and north-west periphery of the city. The city of Chicago shows intermediate patterns of biophysical and social connectivity across the garden network, with a small bias towards low connectivity, but a more even distribution of connectivity beyond these areas of dearth (Fig. 2). Socio-ecological connectivity across the network was moderate across Chicago, except for one highly-connected cluster on the west side of the city (Fig. 2c). New York City has areas of high socio-ecological connectivity that contrast with expanses of very low connectivity across its network (Fig. 3), leading to a more bimodal pattern of connectivity (Fig. 4). New York City’s boroughs of Manhattan, south Bronx, and north Brooklyn were the primary areas of high socio-ecological connectivity in their urban garden networks. Northern Bronx and western Queens have moderate-low socio-ecological connectivity, with less socio-ecological connectivity on the eastern side of Queens, and very little socio-ecological connectivity in Staten Island (though this is likely due to few gardens in that borough).

**Figure 5:**
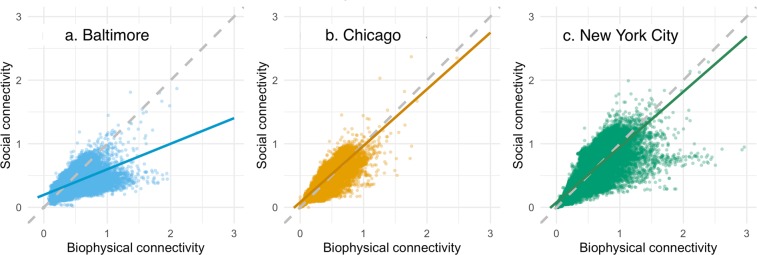
The relationship between biophysical and social connectivity illustrated for each city: Baltimore (**a**), Chicago (**b**), and New York City (**c**). Here, the dashed line (at slope (m) = 1) represents a theoretical equal contribution of the biophysical and social connectivity across the landscape. The deviations from that line, indicated by the solid line fitted to the data distributions, suggest the degree to which connectivity is biased towards one type of connectivity. (Relationship is not assumed linear, see text).

The relative strength of the biophysical versus social connectivity differed in each city (Fig. 5). Here, while we do not assume a linear relationship between biophysical and social connectivity, we can compare the ratio of each component to the linear slope (i.e. m = 1) to assess whether standardized biophysical or social connectivity is higher in each city. Thus, socio-ecological connectivity in Baltimore is a result of higher biophysical connectivity. Alternatively, socio-ecological connectivity in Chicago and NYC is comprised of relatively equal biophysical and social features. In NYC and Baltimore, median biophysical connectivity was slightly higher than median social connectivity.

### Hotspots and coldspots in the landscape

**Figure 6:**
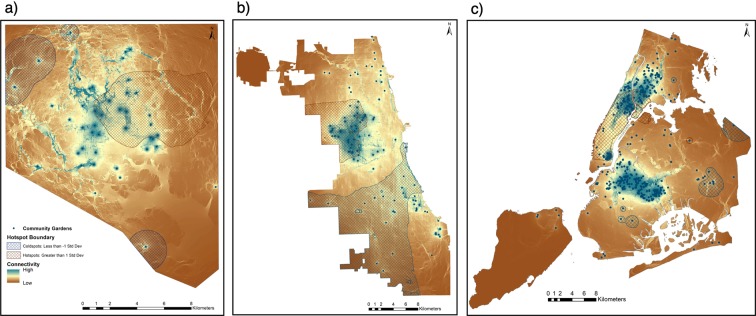
Total cumulative density for Baltimore (**a**), Chicago (**b**), and NYC (**c**) with areas (500 m evaluation distance) of clustered or isolated gardens determined by a Hotspot Analysis employing the Gi* spatial statistic to analyze spatial dependency in terms of density or clustering of features within a specified area. Red hatch overlays represent hotspots (>1 SD); blue hatch overlays represent coldspots (<−1 SD). Nodes with no surrounding connectivity did not have a pairwise match. Maps produced using NLCD and NAIP satellite imagery data in ArcGIS (Table 3).

Across all cities, hotspots tended toward areas of higher overall connectivity, whereas coldspots included any statistically significant garden clusters that were isolated from other gardens in the network. The cities differed in the number, size, and distribution of hot and coldspots of garden locations in their networks across the landscape (Fig. 6). Specifically, each city had at least one relatively large hotspot that encompassed many gardens. The majority of Baltimore’s area of high connectivity – just northeast of the CBD – was a significant hotspot. Baltimore had one coldspot south of the CBD and one in the northwest suburbs (e.g. Central Park Heights area). Chicago had one hotspot located on the West Side, and one large coldspot that encompassed a spread of gardens distributed across the south and southwest sides of the city. In NYC, a hotspot primarily encompassed the majority of Manhattan, extending into Bronx and Brooklyn. NYC had five coldspots, each encompassing one to six gardens in the network. To compare results across cities, Baltimore and NYC coldspots tended toward the city edge and fringe of the city’s boroughs.

## Discussion

This study illustrates how social-ecological systems can create networks of biophysical and social connectivity across urban landscapes, but also how heterogeneity in biophysical and social features of urban landscapes create different connectivity networks. Furthermore, our hot- and coldspot analyses provide evidence that the flows of ecosystem services across these networks are uneven across the landscape, and indicates modularity in the network. This may have tangible effects on life within these areas, and the potential for areas to be resilient to environmental change or political economic change. Though most connectivity analyses at large spatial extents focus on structural landscape connectivity exclusively from a biophysical perspective^35,80,82^, we argue for considering how both biophysical and social features collectively shape the ability of socio-ecological networks to support flows at the landscape scale. This is underpinned by the observed differences in the magnitude and directionality of socio-ecological connectivity among the cities. Such interdisciplinary analyses with applied outcomes can provide new organizational and planning tools to stakeholder groups, and to conservation planning and city regional planning to promote urban ecological and social connectivity and resilience.

### Landscape features shape socio-ecological connectivity flows

Our first question was how gardens create a theoretical network of biophysical and social connectivity across different urban landscape contexts. Community gardens are an ideal model system to investigate this question because they are social-ecological systems: they support social interactions between people, and ecological interactions between abiotic and biotic environmental features. We show that, in theory, these gardens have the capacity to create a broad network of socio-ecological connectivity, which can support ecosystem service flows across urban landscapes^83^. However, these patterns in total connectivity differed between our three cities. In terms of median connectivity, Baltimore has a more even distribution, while NYC – and to a lesser extent, Chicago – show significant zero inflation. These similarities between NYC and Chicago connectivity patterns likely derive from the significant clustering patterns in garden distribution in these cities^84^. The clusters in NYC support the highest values of connectivity across our three landscapes — showing that these clusters exhibit high modularity via internal interactions that generate high internal connectivity. However, our model indicates that it is difficult for connectivity of ecosystem service flows to extend beyond these tight clusters, particularly in densely built NYC neighborhoods. The connectivity and potential resilience benefits of network modularity may therefore be limited to the neighborhood. Thus, the disparate pattern produced through network modularity suggests that there may be scale-dependent trade-offs in neighborhood versus city-scale ecosystem services provided by community gardens and merits further investigation^45^. While Chicago gardens are also significantly clustered, the combination of a less-dense city with more green space than NYC and more gardens nodes than Baltimore results in a unique pattern of connectivity across the landscape where there is a slight zero-inflation but overall a fairly moderate distribution of connectivity.

NYC and Chicago are also distinct from Baltimore in that these cities have relatively higher contributions of social connectivity to the overall patterns on the landscape. Clustered gardens which facilitate high social connectivity may reflect deliberate acts to increase food security and sense of community in neighborhoods of low income and minority populations. In NYC, gardens were often implemented to build social cohesion in and provide ecosystem services to low income communities and minority communities seeking social integration^42,53,85^. This supports the idea that neighborhood scales where people interact most closely with one another may be more important than the city scale for these interactions in NYC and Chicago. However, in less-dense cities like Baltimore which have more green spaces and considerably higher biophysical connectivity, gardens may be links in broader ecological networks that include yards, parks, and other green spaces. There are some high connectivity flows along the Chicago state border – a critical biodiversity hotspot home to endemic species and habitats in the region and a priority site for socio-ecological restoration^85^. Implementing small and novel urban habitats such as gardens in neighborhoods in this region might connect with natural areas in the landscape to support broader conservation goals of the city and benefit garden regulating services such as pollination through habitat connectivity^86,87^.

### Hotspots and coldspots in the landscape can inform opportunities

We investigated hotspots and coldspots of garden clusters in the landscape relative to socio-ecological connectivity to inform potential garden placement to improve landscape-scale connectivity for urban resilience. Similar analyses have been used to improve public health and transportation^88^. We found that hot and coldspot patterns were city specific, and application to city planning must be treated so. To take the case of NYC, the clustering of gardens in NYC creates a spatially large hotspot with moderate to high connectivity among gardens, suggesting that these gardens interact strongly with one another to promote socio-ecological connectivity in these neighborhoods and adjacent neighborhoods. This suggests high modularity of gardens in NYC neighborhoods, where clusters of gardens across the network interact strongly with one another and are thus highly internally connected. While the implications of modularity on overall network resilience are unclear, to improve connectivity of ecosystem services and bolster a resilient gardening network across NYC, future garden development in the network may be most effective in neighborhoods that utilize existing moderate connectivity to begin to bridge gaps between hot and cold spots across the city. Neighborhoods that meet these criteria might include the eastern portion of the Bronx and Queens and the southern portion of Brooklyn. Staten Island is sufficiently isolated as an island and does not gain from connectivity and clustering produced and shared across the other boroughs. In Baltimore, the coldspot in the peninsula and at the fringe of the city is expected due to the lack of other gardens in the vicinity, and may suggest to conservation planners for example, that establishing gardens or greenways in these isolated areas can better connect a fragmented landscape through green space or garden linkages to broaden connectivity flows. In Chicago, the relatively consistent spread of moderate connectivity may relate to the deliberate presence of large and small urban parks (which typically do not contain community gardens) throughout Chicago, which is a legacy of design priorities in the nineteenth century^89^. Chicago has a significant coldspot in the area where connectivity between gardens dwindles and gardens become more spread out, suggesting that garden implementation in the southwest region of the city may contribute to additional connectivity. In sum, our goal here is not necessarily to make specific recommendations for these cities (as the models are dependent on theoretical and methodological assumptions rather than historical-political context), but rather to highlight this type of analysis as a useful tool. Stakeholders can examine maps and contextualize the results in their firsthand knowledge of the landscape. Although here we use garden establishment as a possibility, this type of analysis could effectively be tailored to a city or to other forms of green spaces or social spaces with connectivity and urban resilience in mind by both conservation planners and city planners^90,91^.

### Study limitations and future directions

Our study provides fodder for future research in social and ecological landscape connectivity analysis. The method of biophysical connectivity assumes similar local habitat features to focus on the landscape scale, and future research could measure and incorporate the local features of the habitat relevant to mobile ecosystem service providers (e.g., canopy cover, plant diversity, floral availability) in models. The method of social connectivity in this study focuses on a particular set of social features, and future studies could focus on other forms of social connectivity important to social mobility (e.g., transportation networks, telephone lines, etc.) in a particular city or social-ecological system context. Our analysis is limited by the geopolitical boundary of each city, but ecological – and to a lesser extent social – processes are not limited by arbitrary spatial demarcations because external landscape features may influence connectivity flows. Areas for future research include validating models with field collected data, exploring modularity versus dispersed connectivity in relation to resiliency, assessing spatial scale-dependent patterns, expanding research to other cities and across time periods, and using other analytical tools such as centrality analysis^70,92^ to identify areas to add nodes for enhancing connectivity.

## Conclusion

Social-ecological systems such as community gardens can promote landscape-scale ecosystem service flows through socio-ecological connectivity. But the nature of these flows (e.g., magnitude, direction) change with landscape biophysical and social heterogeneity. This may have important implications for how human populations benefit from ecosystem services and the resiliency of urban landscapes. The work presented in this study contributes to a growing body of work in spatial landscape ecology that incorporates both structural and functional connectivity to assess how multiple features of the landscape create and influence a network across that landscape^73^. The methods employed here can contribute to urban planning by incorporating conservation planning and moving focus to improving green infrastructure connectivity through garden development to benefit society and the built environment^70,93^. In demonstrating how connectivity differs across a city and highlighting hot- and coldspots in the landscape, this study provides an example of how connectivity analyses could be harnessed by local stakeholders to make informed land use planning decisions to build urban resilience.

## Methods

### Theoretical background of connectivity and its measurement

Connectivity across a landscape measures the degree to which the landscape facilitates movement between resource patches or “nodes”^24^. There is relatively higher conductance of connectivity when organisms can easily disperse between nodes across the landscape, and higher resistance to connectivity in the landscape when barriers prevent organisms from easy dispersal between nodes across the landscape. New analytical approaches use metrics to account for connectivity across nodes in the landscape that create networks or corridors facilitating organisms’ movement^94–97^. These metrics typically leverage graph theory to estimate landscape resistance between nodes^98^. One extension of graph theory that models pairwise relationships between objects is based on electrical circuit theory and provides a robust method to quantify movement across multiple paths using the concepts and metrics of electrical circuits. The method facilitates the prediction of multiple conduits by accounting for both structure (i.e., landscape pattern) and function (i.e., organism movement and interaction) of the landscape by calculating the “random walk” between pairs of nodes (using resistance and distance to the node and back), resulting in a continuous connectivity surface incorporating all potential movement into the measure of resistance^99^. These theoretical foundations provide circuit theory a firm grounding in the context of landscape-scale movement ecology^79,94^.

“Hotspots” of high connectivity can arise in the landscape when there is high density or spatial clustering of nodes. Hotspots indicate modularity within a network. Conversely, where there is low density and clustering, “coldspots” can arise in the landscape. Distance statistics can analyze the spatial association (density, clustering) and spatial dependency within a user area^82^ (described in detail below). In this study, hotspot and coldspot analyses are relevant – especially to stakeholders – because they can identify areas of garden saturation or isolation relative to level of connectivity within the network where, for example future urban garden establishment may improve biophysical and social connectivity across the network and thus landscape-scale network resilience, or where stakeholders may examine benefits of and further support network modularity.

### Network nodes

We theorize community gardens as nodes of ecosystem service flows because they are centers of ecological and social processes. In each of the three cities, we collaborated with a well-established stakeholder group that support community gardens to obtain the spatial locations of gardens: Baltimore Green Space in Baltimore, MD (n = 43 gardens); Green Thumb in NYC, NY (n = 476); NeighborSpace in Chicago, IL (n = 116). We used a stakeholder-informed approach rather than attempting to locate all possible gardens because the stakeholder gardens are (1) known to exist, (2) more likely to persist over time, and (3) provided a standardized, generalizable approach. We acknowledge that this approach does not account for all gardens in these city landscapes, nor does it account for the habitat size or features within a garden site that may influence, for example biodiversity of service providers. The process of spatial mapping and validation of garden presence is described elsewhere^100^. We assigned the geographic centroid of each city-sanctioned, organization-supported urban garden to be a node in the analysis. Here, the level of social-ecological connectivity and flow are supported by the activity of the stakeholder as stewards of urban ecosystem services.

### Resistance landscapes

Resistance can be theorized as the hindrance of movement across the city landscape^101,102^. Here, we assessed the social and biophysical resistance of the landscape to connectivity around gardens. We built two spatially-continuous theoretical resistance models for all three cities based on biophysical and social features that: (1) represents the theoretical degree of environmental resistance posed by land cover features to the flow of ecosystem services; and (2) represents the theoretical degree of socio-cultural and economic resistance to flows of human well-being posed by social features (Table 3).

**Table 3:** Biophysical and social variables used to build the resistance landscapes (both resistance base and resistance reduction variables) with the justification for their use and the data source, respectively.

Analysis	Variable (buffer)	Data source
**Social Resistance Base**	^†^Proportion of Black and African American residents^116^	US Census Bureau^115^
	Proportion of Hispanic residents^117^	
	Total housing	
	Total population/ Population Density^118^	
	Average Household Size^119^	
	Proportion of households with children under 18^119^	
	Median household income^118^	
	Proportion of renters^118^	
	Proportion of vacant properties^116,120^	
	Median year structure was built^118^	
	Median age^119^	
	Median cost of rent^121^	
**Social Resistance Reduction** (**buffer distance**)	Public Schools (500 m)^122^	Esri Data & Maps^123^ **Baltimore specific**: City of Baltimore^124,125^ **Chicago specific**: City of Chicago^126,127^ **New York City specific**: City of New York^128,129^
	City Parks (250 m)^118,130^	
	Places of Worship (500 m)^131,132^	
	Community Centers (250 m)^133,134^	
	Libraries (250 m)^135,136^	
	Access to Food^137^	USDA Economic Research Service^138^
	Crime Index <200 (50 m)^118,119,139^	ESRI^140^
	Human Health Index (highest quartile)^141^	ESRI^142^
	Properly permitted and non-contaminated EPA sites	§US Environmental Protection Agency (EPA)^143^.
**Biophysical Resistance Base**	LiDAR and NAIP satellite imagery from the University of Vermont Spatial Analysis Laboratory	University of Vermont^104^
	National Land Cover Database (NLCD) Development Categories (classes # 21, 22, 23, 24)	NLCD^103^
**Biophysical Resistance Reduction** (**buffer distance**)	Properly permitted and non-contaminated EPA sites	US Environmental Protection Agency^143^
	US Federal conservation lands (500 m buffer)	US Geological Survey^144^
	Wetlands (500 m buffer)	US Fish & Wildlife^145^
	Large green spaces (grass cover > 2500 m^2 ^)	University of Vermont^104^

Note that sociodemographic variables are used as defined by the US Government’s Census Bureau^115^ and are at the scale of the Census tract.

^†^According to the US Census Bureau, “Black or African American” refers to a person having origins in any of the Black racial groups of Africa. The Black racial category includes people who identified as “Black, African Am., or Negro” and who identified as African American, Sub-Saharan African, and Afro-Caribbean^108^. ^§^Geospatial information for all publicly available FRS facilities that have latitude/longitude data.

To create the biophysical resistance model, we collected high resolution land cover imagery for each city from publicly available databases. We used the 2011 National Land Cover Database^103^ for data on building typology (land cover classes #21–24). We used rasterized LiDAR and National Agriculture Imagery Program (NAIP) satellite imagery from the University of Vermont Spatial Analysis Laboratory for data on built versus natural cover^104^ (Table 3). These spatial data classify tree canopy cover, grass/shrub cover, bare soil, standing or open water, buildings, roads/railroads, and other paved surfaces at a 3 × 3 m spatial (pixel) resolution. Chicago imagery was provided at 1 × 1 m resolution but, to afford computational capacity and consistency across cities, was scaled to 3 × 3 m. Land cover resistance was modified with correction factors for: permitted facilities in compliance with Environmental Protection Agency (EPA) regulations; conservation lands (federal and wetlands); and grass patches >2,500 m^2^ to capture natural or open space areas other than private lawns and parks (Table 3). We assigned a 10% reduction of the base resistance to these factors, and where appropriate, buffers were applied to capture biophysical effects that occur beyond a particular point (Table 3). Because no reduction value is standard for connectivity analyses, we chose a 10% reduction value as an experimental value that could be generalized for future studies. The final resistance landscape ranges from a resistance of 1 to approximately 180. We note that the range of 1 to 180 is context or study system-dependent, and experimentally determined from the specific variables and scoring system that we selected.

To create the social resistance landscape, we used sociodemographic information from 2011 to 2016 US Census Bureau’s American Community Survey five year Census Block Group estimates for all three cities and selected important variables using a combination of a literature review and empirical statistical tools (Table 3). This approach is predicated on the knowledge that certain socio-demographic factors are known to act as barriers or conduits to social cohesion and connections between people in gardens^28,105,106^. First, we chose census variables *a priori* that impact social connectedness, particularly in relation to community gardening or cultural ecosystem services (Table 3). Three pairs of variables (total housing/total population, average household size/proportion of households with children under 18, median monthly rent/median household income) were highly correlated (Spearman’s Rank Correlations ρ > 0.60) and were therefore excluded from subsequent analyses. Second, we used binary logistic regression models to determine which of these initial variables to include in the social resistance landscape. Because gardens are the focus of this study, and not every block group supports a garden, we considered block groups that contain gardens (“successes” in logistic regression terms) versus those without gardens (“failures”). The selection of variables based on garden locations inserts a level of bias into the social landscape, but this method allowed us to methodically reduce variables to those that have the strongest relationships to community gardening in our focal cities. Furthermore, this approach helped us center our analysis on variables that are directly related to the on-the-ground involvement of the stakeholder groups. Because city-level organizations are known to effectively connect local residents with external resources and protect urban green infrastructure^28^, using the social characteristics of garden locations provides us with indicators of the communities where our stakeholder groups have strong connections and familiarity with residents.

We associated the locations of the community gardens with the Census Block Groups and assessed the presence/absence of community gardens within the Block Group polygons in ArcMap v. 10.4.1. We then used the MuMIn package in R 3.1.1^107^ to run a logistic regression model, assessing a full model of the nine social landscape variables of interest. We applied an Akaike Information Criterion (AIC) model selection using the “dredge” function (where lower AIC values indicate a better model fit), and model-averaged the models that combined to explain 97% of the variation in the dataset (∑ ω = 0.97). We then calculated variable importance weights for the nine target variables. Six variables in the analysis had a variable importance weight of 1.00, meaning that they were in every model that contributed to a 0.97 confidence model set: average household size, median age, the proportion of Black and African American residents^108^, the proportion of Hispanic residents, proportion of renters, and proportion of vacant properties. We used all six variables in the social resistance surface (Table 3).

We calculated standardized regression coefficients for each variable and assessed the directionality of the relationship between each of these variables and the changes in likelihood of having a community garden in the given block. To assign resistance values to the social landscape based on this directionality, we divided the 180 maximum resistance value from the biophysical landscape among the six social variables for comparable minimum and maximum values for both resistance landscapes. We divided the values of each variable into three equal groups, and assigned resistance values of 10, 20, or 30 to these groups based on the direction of the relationship (i.e. the sign of the regression coefficient for each variable). This provided a base for the social resistance and ranged from a resistance of 1 to 180 (comparable to the biophysical resistance). To note, the base and final resistance have the same range (1 to 180) because pixels may experience no resistance reduction (180) or experience a range of reductions (>180). The social resistance was modified with correction factors and appropriate buffers for attributes of the landscape that promote human well-being and rely on or inspire community cohesion (Table 3). Buffers (1 km) were applied around the city boundaries for biophysical and social resistance surfaces to minimize edge effect on the final connectivity surface.

### Landscape connectivity

To identify areas with high predicted biophysical and social connectivity among gardens, we used the software GFlow^109^, a software that provides flexibility in modeling connectivity over multiple scales and extents by removing the limitation of computational restrictions through the use of parallelization of computations^109,110^. GFlow expands the capacities of Circuitscape (see http://www.circuitscape.org; the most widely used landscape connectivity software) to significantly larger extents and finer scales^109,110^. These improvements provided the capacity to calculate fine-scaled biophysical and social resistance surfaces over many iterations at varying spatial scales depending on the landscape or parcel-based analysis. Current densities within the resistance grid cells are measured as current moves through the resistance surface between garden nodes, creating weighted current density sums across all pairs of nodes in the model (see Table 1 for explanation of current). Output assumptions can be changed such that individual pairs can be evaluated if necessary. Both sheet flow — the diffusion of current across the landscape, and dense flow (also called pinch-points) — flow guided by high resistance surface areas channeling flow, are flow characteristics generated from the models.

We analyzed each study area by connecting source and destination locations in a pairwise fashion, using a convergence factor (convergence of current from two sources over a number of iterations to a specified level of accuracy, here four decimal digits). These locations represent the centroid of a polygon garden patch. Utilization of the supercomputing approach allowed us to run the analyses simultaneously across the model’s extent. The model output contained a surface and table of the summation of per-cell current density (in amperes) for a random selection of pairwise nodes. Models were produced at a 3 m output resolution for the three study areas to summarize cumulative density between random pairs of nodes across each city’s biophysical and social resistance surface.

### Analysis of connectivity magnitude and direction

To compare the magnitude of overall connectivity between the three cities, we used the Random Points tool in ArcGIS v. 10.5^111^ to select 50,000 random points from within the city limits. For all points that had data (i.e., did not fall in waterways or other unmapped areas), we extracted the current density values for social and biophysical layers (n_balt_ = 49,991, n_chi_ = 42,376, n_nyc_ = 40,426). We normalized these data on a scale of zero to one for each layer and summed them to get a final connectivity value that ranged from zero to two. We square-root transformed the normalized score to adjust for the zero-inflated structure of the data. We used the square-root transformed connectivity score to construct a density graph for each city and extracted summary values to describe and compare connectivity patterns between cities.

To determine where socio-ecological connectivity is high due to high contributions of both social and biophysical connectivity, we extracted locations where biophysical and social connectivity were independently greater than one standard deviation of the mean of the respective biophysical or social layer. We then used the “Extract by Mask” tool in ArcGIS 10.4.1^111^ to identify areas where high biophysical connectivity and high social connectivity overlapped. This allowed us to determine spatially where combined socio-ecological connectivity was highest.

To determine how social and biophysical characteristics influence the connectivity profile of each city, we used log-transformed normalized connectivity values for both layers at the randomly sampled points used in the connectedness evaluation. We compared the slope of the ratio between standardized biophysical and social connectivity to a null line of m = 1 for each city to compare the relative values of social and biophysical connectivity in each city. Here, we are not working under the assumption that there is a linear relationship between biophysical and social connectivity, but are instead comparing their relative contributions to the total socio-ecological connectivity of the landscape. A slope of one indicates equal biophysical and social connectivity, while deviations in either direction (i.e., significantly greater or less than a slope of one) indicate the relative weight of one in comparison to the other. The intercept of this biophysical:social connectivity ratio also allows us to begin to compare the overall connectivity patterns between the cities. Notably, our goal in this analysis is not to comprehensively model the relationship between biophysical and social connectivity. Rather, we are interested in providing initial insight into the nature of this relationship in our system and laying the foundations for future research.

### Analysis of hotspots and coldspots

To identify significant “hotspots” and “coldspots” of garden clusters in the landscapes based on the density of the gardens within an area, we performed a hotspot analysis using the Hot Spot Analysis tool in ArcGIS v. 10.5^111^. Hotspot analyses jointly evaluate density or clustering of features within a specified area, and employs the Getis Ord Gi* (Gi*) spatial statistic to analyze spatial dependency in terms of frequency and attribute values within a defined spatial framework. The results of the analysis are given in terms of Z-scores and p-values that identify the statistical significance of individual collections or clusters of gardens. Areas with a large Z-score and small p-value indicate a hotspot of clustering (and typically connectivity); coldspots are features with small or negative Z-scores and large p-values.

The analysis took into account the density of gardens within a fixed distance (500 m grids) across a given city. This distance was chosen because it is walkable for many people^112^, accommodates flight distance for resident and migratory birds, and is a limit for pollinator movement in urban areas^113,114^. Using the ArcGIS toolset reference for the spatial statistics toolbox as a general methodology template, garden points were aggregated and evaluated for statistical significance (greater than one standard deviation from the median). Finally, inverse distance weighting was run to interpolate a raster surface of hot- and coldspot for each city. This surface provided a means to identify where hot and coldspots are not only in each city, but also in reference to the individual gardens and to the connectivity surfaces.

## Data Availability

All data and analysis code will be made available upon reasonable request, and will be deposited in Dryad upon manuscript publication.
